# Supplementary material for: Complexome profiling of the Chlamydomonas psb28 mutant reveals TEF5 as an early PSII assembly factor
Source: Plant Cell. 2025 Mar 17;37(6):koaf055. doi: 10.1093/plcell/koaf055 (PMC12164590; doi:10.1093/plcell/koaf055)
Supplement: koaf055_Supplementary_Data [file koaf055_supplementary_data.zip › psb28_interactive.zip!/plots/intensity/qualityPlots/peptides/_Cre16.g668600.t1.2.html]

### Cre16.g668600.t1.2

Trivial name:   
SeqCoverage [median]: 25.50  
Mass: 35.01 kDa  
  
 

| Sequence | MissedCleavage | Mass [Da] | wtIntensity | muIntensity | Score |
| --- | --- | --- | --- | --- | --- |
| AEELGVTR | 0 | 873.46 | 1487.23 | 3062.09 | 140.09 |
| FVVDTFK | 0 | 854.45 | 2992.90 | 0.00 | 97.07 |
| GLEALQTVR | 0 | 985.56 | 4137.02 | 1962.38 | 94.31 |
| GQAVVVTEVAEGSR | 0 | 1400.73 | 1491.48 | 4188.17 | 119.45 |
| LGLVPDGR | 0 | 825.47 | 1174.02 | 0.00 | 81.30 |
| MNALSDPIR | 0 | 1015.51 | 0.00 | 750.58 | 67.99 |
| NQPIDLQFEPMMK | 0 | 1589.76 | 568.84 | 1559.46 | 85.81 |
| VAVNQVQK | 0 | 884.51 | 3031.43 | 3235.62 | 187.14 |
| YQEQQR | 0 | 850.39 | 594.02 | 448.08 | 128.38 |
|  |  |  | 15476.9 | 15206.4 |  |
